# Bone health and awareness of osteoporosis in women aged 40 to 60 years in Jiaxing City, China

**DOI:** 10.1097/MD.0000000000038073

**Published:** 2024-05-10

**Authors:** Zhengfen Xu, Ying Zhou, Xiaojie Wu, Huan Li, Wei Bian

**Affiliations:** a Department of Gynecology, Jiaxing Maternity and Child Health Care Hospital, College of Medicine, Jiaxing University, Jiaxing, China; b Department of Nursing, Jiaxing Maternity and Child Health Care Hospital, College of Medicine, Jiaxing University, Jiaxing, China; c Department of Radiology, Jiaxing Maternity and Child Health Care Hospital, College of Medicine, Jiaxing University, Jiaxing, China.

**Keywords:** awareness, bone health, osteoporosis, Osteoporosis Prevention and Assessment Tool (OPAAT)

## Abstract

The objective of this study is to evaluate the pattern of bone mineral density (BMD) in native Jiaxing women, and to investigate their awareness of osteoporosis. A total of 538 native Jiaxing women aged 40 to 60 years were recruited from January 2022 to December 2023 when they had routine examinations in the physical examination center of Jiaxing Maternal and Child Health Hospital. The Chinese version of Osteoporosis Prevention and Cognition Tool was used to evaluate participants’ cognitive level of osteoporosis. BMD of participants’ lumbar spine (L1–L4) and left hip (Neck/Troch/Ward) was measured by dual-energy X-ray absorptiometry. The mean total score of the awareness about osteoporosis (general knowledge, complications, and prevention) was 22.08 ± 2.74, which was suboptimal. The higher the education level, the higher the score of awareness (*P* < .01). Medical staff had the highest awareness rate of osteoporosis and the farmer had the lowest. Lumber spine and hip BMD of all sites was significantly decreased with increasing age (*P* < .001). Premenopausal women had higher BMD than postmenopausal women at all lumbar spine and hip sites (*P* < .01). The overall frequency of osteoporosis was 10.8% in the lumbar spine, 8.6% in the total hip, and 17.7% in either site. Osteoporosis and osteopenia are highly prevalent among native Jiaxing women but their awareness of osteoporosis is inadequate. To reduce the prevalence of osteoporosis, especially among the unemployed, we should carry out effective health education through multimedia to raise their awareness of osteoporosis. In addition, menopausal hormone therapy should also be considered in menopausal women.

## 1. Introduction

Osteoporosis is a common systemic skeletal disease and frequently affects middle-aged and elderly people, which seriously endangers postmenopausal women health and increases healthcare costs. The disease is characterized by low bone mass and microarchitectural deterioration of bone tissue, which can easily lead to fracture and has become one of the serious social problems in the world today.^[1]^ According to the epidemiological survey of osteoporosis in China, the prevalence of osteoporosis in women over 50 years old is 32.1%, and 51.6% in women over 65 years old.^[2]^ Patients with osteopenia and early osteoporosis usually have no obvious clinical symptoms. Due to a lack of awareness of osteoporosis, most of the women do not take timely intervention measures in the early stage of bone loss, thus delaying the optimal diagnosis and treatment period.

According to the WHO criteria, the diagnosis of osteoporosis was based on SD scores between bone mineral density (BMD) and peak bone mass in healthy young women. Osteoporosis is defined as a T-score (BMD score) of −2.5 or lower and osteopenia is defined as a T-score between −1 and −2.5.^[3]^ In women, BMD decreases sharply in the perimenopausal period because of loss of estrogen (age 40–60 years).^[4,5]^ Osteoporosis awareness and early diagnosis are important for preventing fractures and deducting associated economic burden. In 2022, China proposed a special action, requiring local governments to actively carry out epidemiological surveys to understand the bone health of residents. Jiaxing City is located in the Yangtze River Delta, with more cloudy, rainy days, less sunshine, and many women like to work indoors and spend relatively less time basking in the sun. To date, there are very few studies evaluate the pattern of BMD and the awareness of osteoporosis among native Jiaxing women. Therefore, we conducted the study in native Jiaxing women aged 40 to 60 years. It is hoped that the findings of the study will help the prevention and control of osteoporosis in native Jiaxing women.

## 2. Methods

### 2.1. Subjects

A total of 538 native Jiaxing women aged 40 to 60 years were recruited from January 2022 to December 2023 when they had routine examinations in the physical examination center of Jiaxing Maternal and Child Health Hospital. The exclusion criteria were as follows: Use of hormones or drugs that affect bone metabolism; Has been diagnosed as osteoporosis or osteopenia; Intellectual disability, unable to communicate normally and complete the questionnaire independently; Unable to tolerate BMD examination; pregnant woman. This study was approved by the ethics committee of Jiaxing Maternity and Child Health Care Hospital (No. 2019/066). All participants signed an informed consent form.

### 2.2. Questionnaire

We use the Chinese version of the Osteoporosis Prevention and Cognition Tool (OPAAT-C) to evaluate participants’ cognitive levels of osteoporosis in this study. It has been proved the OPAAT-C has good reliability and good psychological evaluation indicators and it can be used as a validated tool to assess the level of osteoporosis awareness of the Chinese public.^[6]^ The questionnaire consists of 30 questions, including 3 parts: general knowledge of osteoporosis, complications of osteoporosis, and prevention of osteoporosis. Each question has 3 answers: correct, incorrect and I do not know. The scoring rule is: 1 point for correct answer, 0 points for the wrong answer and I do not know. The total score ≥24 is high knowledge, 19 to 23 is general knowledge, and ≤18 is low knowledge. One training nurse gathered and checked the questionnaire and characteristic about the participates including age, menopausal status, education level, occupation, past medical history and use of medications one by one. If there is any missing item in the questionnaire, it will be regarded as invalid.

### 2.3. BMD examination

BMD of participants’ lumbar spine (L1–L4) and left hip (Neck/Troch/Ward) was measured by dual-energy X-ray absorptiometry produced by Hologic Discovery (Boston, America). The parameters are expressed in BMD (g/cm^2^).

### 2.4. The diagnose of osteoporosis and osteopenia

The peak BMD and standard deviation of Chinese women obtained from the 2018 China Epidemiological Survey of Osteoporosis were used as background reference values to calculate the T value.^[2]^ T = (corrected BMD measurement value − peak bone density of normal adults of the same sex and race)/(standard deviation of BMD of normal adults). The instrument was calibrated first after starting every day, and the measurement was carried out by a specially assigned person. According to the WHO criteria, osteoporosis is defined as a T-score (BMD score) of −2.5 or lower and osteopenia is defined as a T-score between −1 and −2.5.^[3]^

### 2.5. Statistical analysis

Questionnaire data were double entered into Excel (Microsoft Excel 97-2003) and analyzed in SPSS Statistics 25 (IBM, New York, NY). First data was checked for normal distribution using Kolmogorov-Smirnov test. Continuous variables meet with normal distribution were reported as mean ± standard deviation (SD). *t*-test was used for comparison between the 2 groups, and one-way ANOVA was used for comparison between multiple groups. Categorical variables were reported as numbers (N) and proportions (%). The frequencies of categorical variables were compared using Pearson Chi-square or Fisher exact test. *P* < .05 was considered statistically significant.

## 3. Results

### 3.1. Sociodemographic characteristics of the 538 Jiaxing native women with OPAAT scores

A total of 538 women who met the qualification criteria agreed to participate in the study and completed the questionnaire. The average age was 50.11 ± 4.54. Most women were in premenopausal stage (61.3%) and 38.7% of the subjects were in post-menopause stage. The average total score of awareness about osteoporosis (general knowledge, complications, and prevention) was 22.08 ± 2.74, which is only a fair level. According to the comparison of the score of the 3 parts by demographic status, there is no differences for ages and menopause status were found. The higher the education level, the higher the score of each part (*P* < .01). Medical staff had the highest awareness rate of osteoporosis and the farmer had the lowest (Table 1).

**Table 1:** Sociodemographic characteristics of 538 Jiaxing native women with OPAAT scores.

Variable	Categories	*N* (%)	General knowledge (11)	Complications (5)	Prevention (14)	Total score (30)
Age						
	40–45	144 (26.7%)	8.53 ± 1.39	2.95 ± 0.68	10.95 ± 1.73	22.43 ± 2.66
	46–50	129 (24%)	8.46 ± 1.34	2.87 ± 0.74	10.44 ± 1.63	21.77 ± 2.55
	51–55	171 (31.8%)	8.55 ± 1.38	2.87 ± 0.80	10.57 ± 1.97	21.99 ± 2.83
	56–60	94 (17.5%)	8.37 ± 1.31	3.00 ± 0.87	10.76 ± 1.71	22.13 ± 2.90
	*F*		0.42	0.88	2.14	1.46
	*P* for trend		.74	.45	.09	.23
Menopause status						
	Yes	208 (38.7%)	8.51 ± 1.33	2.9 ± 0.68	10.70 ± 1.72	22.4 ± 2.6
	No	330 (61.3%)	8.47 ± 1.40	2.93 ± 0.89	10.75 ± 1.89	22.14 ± 2.95
	*t*		0.13	0.23	0.23	0.09
	*P* value		.72	.63	.60	.76
Education						
	Primary school or less	94 (17.5%)	7.62 ± 1.39	2.62 ± 0.93	9.59 ± 1.87	19.82 ± 2.59
	High school	66 (12.3%)	8.14 ± 1.35	2.67 ± 0.77	10.44 ± 2.18	21.24 ± 2.95
	Training school	131 (24.3%)	8.58 ± 1.30	2.9 ± 0.79	10.9 ± 1.65	22.37 ± 2.40
	College degree or high	247 (45.9%)	8.88 ± 1.20	3.10 ± 0.63	11.03 ± 1.53	23.00 ± 2.32
	*F*		24.04	12.31	17.49	40.91
	*P* for trend		0.000	0.000	0.000	0.000
Occupation						
	Farmer	56 (10.4%)	8.36 ± 0.93	1.57 ± 1.01	8.07 ± 1.59	18.00 ± 2.04
	Housewife	48 (8.9%)	8.38 ± 1.26	2.71 ± 0.68	10.04 ± 1.90	20.1 ± 2.35
	Retired	45 (8.4%)	8.47 ± 1.53	2.91 ± 0.93	10.60 ± 1.74	21.80 ± 2.79
	Employee	262 (48.7%)	8.54 ± 1.35	2.97 ± 0.73	10.69 ± 1.75	22.22 ± 2.61
	Medical staff	127 (23.6%)	8.90 ± 1.14	3.00 ± 0.67	11.19 ± 1.55	23.09 ± 2.43
	*F*		12.62	13.42	12.58	21.45
	*P* for trend		.000	.000	.000	.000

OPAAT = osteoporosis prevention and assessment tool.

### 3.2. BMD of 538 Jiaxing native women at all lumbar spine and hip sites

Lumber spine and hip BMD of all sites was significantly decreased with increasing age (*P* < .001). Premenopausal women had higher BMD than postmenopausal women at all lumbar spine and hip sites (*P* < .01) (Table 2).

**Table 2:** BMD of the lumbar spine and the total hip of 538 native Jiaxing women aged 40 to 60 yr old.

Variable	N (%)	L1	L2	L3	L4	L1–L4	Neck	Total hip
Age								
40–44	144 (26.7%)	0.90 ± 0.11	0.95 ± 0.11	1.0 ± 0.11	0.98 ± 0.13	0.96 ± 0.12	0.76 ± 0.11	0.87 ± 0.11
45–49	129 (24%)	0.89 ± 0.12	0.94 ± 0.12	0.99 ± 0.14	0.95 ± 0.12	0.94 ± 0.12	0.73 ± 0.12	0.84 ± 0.12
50–54	171 (31.8%)	0.87 ± 0.11	0.92 ± 0.13	0.95 ± 0.13	0.94 ± 0.10	0.92 ± 0.12	0.71 ± 0.10	0.83 ± 0.11
55–59	94 (17.5%)	0.80 ± 0.16	0.84 ± 0.15	0.86 ± 0.15	0.86 ± 0.16	0.84 ± 0.15	0.63 ± 0.09	0.73 ± 0.10
*F*		15.44	17.65	20.41	13.86	18.45	31.70	32.61
*P* for trend		＜.001	＜.001	＜.001	＜.001	＜.001	＜.001	＜.001
Status of menopause								
No	208 (38.7%)	0.89 ± 0.11	0.94 ± 0.12	0.97 ± 0.13	0.96 ± 0.14	0.94 ± 0.12	0.73 ± 0.12	0.84 ± 0.12
Yes	330 (61.3%)	0.83 ± 0.14	0.88 ± 0.14	0.91 ± 0.15	0.91 ± 0.15	0.89 ± 0.14	0.68 ± 0.10	0.80 ± 0.11
*t*		5.2	5.2	5.13	3.45	24.36	4.99	18.68
*P* value		＜.001	＜.001	＜.001	＜.001	＜.001	＜.001	＜.001

BMD = bone mineral density, L = lumbar spine.

### 3.3. Bone health of 538 Jiaxing native women

The overall frequency of osteoporosis was 10.8% in the lumbar spine, 8.6% in the total hip and 17.7% in either the spine or the hip. With increasing age, the frequency of osteoporosis in the lumbar spine, hip or either part also increased. The frequency of osteoporosis for age 56 to 60 years in either the spine or the hip was 38.1% which is only 6.9% for age 41 to 45 years. The frequency of osteoporosis for premenopausal women was 10%, which was 29.8% for postmenopausal women, the difference is significant (Table 3).

**Table 3:** Bone health of 538 native Jiaxing women by age and menopause status.

Variable	Categories	N (%)	Osteoporosis in lumbar spine 58 (10.8%)	Osteoporosis in total hip 46 (8.6%)	Osteoporosis in either part 95 (17.7%)
Age					
	40–45	144 (26.7%)	9 (6.3%)	6 (4.2%)	10 (6.9%)
	46–50	129 (24%)	10 (7.8%)	9 (7.0%)	17 (13.2%)
	51–55	171 (31.8%)	22 (12.9%)	16 (9.4%)	32 (18.7%)
	56–60	94 (17.5%)	17 (18.1%)	15 (16.0%)	36 (38.3%)
*F*			10.29	10.69	40.82
*P* for trend			.016	.014	＜.001
Menopause status					
	Yes	208 (38.7%)	38 (18.3%)	28 (13.5%)	62 (29.8%)
	No	330 (61.3%)	20 (6.1%)	18 (5.5%)	33 (10.0%)
*t*			19.77	10.46	34.43
*P* value			＜.001	.001	＜.001

### 3.4. The level of awareness about osteoporosis according to bone health

More than half of the subjects (56.3%) had fair knowledge of osteoporosis. Only 1/3 of the subjects had high knowledge of osteoporosis. No significant difference in level of awareness was found for the subjects with osteoporosis, osteopenia or normal BMD (Table 4).

**Table 4:** The level of awareness about osteoporosis according to bone health.

BMD results	Low knowledge	Average knowledge	High knowledge	Overall
	64 (11.9%)	303 (56.3%)	171 (31.8%)	(n = 538)
Osteoporosis	17 (17.9%)	44 (46.3%)	34 (35.8%)	95 (17.7%)
Osteopenia	27 (11.5%)	136 (58.1%)	71 (30.3%)	234 (43.5%)
Normal	20 (9.6%)	123 (58.9%)	66 (31.6%)	209 (38.8%)
*F*	4.37	4.72	0.93	
*P* value	.112	.095	.628	

BMD = bone mineral density.

### 3.5. Questions with accuracy rate <50%.

There are 7 questions with accuracy rate <50%. They were: Results in joint pain or swelling of fingers; Results in tooth loss; Glucosamine can help prevent osteoporosis; Everybody will get osteoporosis as it is part of aging; Osteoporosis usually has no symptoms. Weight-bearing exercise (such as brisk walking and line dancing) can decrease bone loss; It is too late to increase calcium intake after age 50 (Table 5).

**Table 5:** Questions with accuracy rate <50%.

Questions	N (%)	
	Correct	Incorrect
Results in joint pain or swelling of fingers. (False)	111 (20.6%)	427 (79.4%)
Results in tooth loss. (False)	124 (23.0%)	414 (77.0%)
Glucosamine can help prevent osteoporosis. (False)	164 (30.5%)	374 (69.5%)
Everybody will get osteoporosis as it is part of aging. (False)	167 (31.0%)	371 (69.0%)
Osteoporosis usually has no symptoms. (True)	240 (44.6%)	298 (55.4%)
Weight-bearing exercise (such as brisk walking and line dancing) can decrease bone loss. (True)	256 (47.6%)	282 (52.4%)
It is too late to increase calcium intake after age 50. (False)	266 (49.4%)	272 (50.6%)

## 4. Discussion

When women reach their mid-40s, bone mass usually begins to decline, and accelerates as estrogen levels decrease after menopause.^[7]^ Our study is consistent with this conclusion that among women aged 40 to 60 years old BMD decreased and the prevalence of osteoporosis increased with increasing age. Moreover, the BMD of postmenopausal women is significantly lower than that of premenopausal women, and the prevalence of osteoporosis in postmenopausal women is significantly higher than that in premenopausal women. Fracture is one of the most common complications of bone loss, leading to disability and death of the patients.^[8]^ Research shows that the prevalence and incidence of osteoporosis fracture is highest in North America and Asia.^[9]^ An estimated 2.7 million hip fractures occurred worldwide in 2010, and 51% of them were preventable if osteoporosis could be avoided.^[8]^ So it is highly recommended to focus our efforts on osteoporosis prevention.

Preventive measures for osteoporosis include raising awareness, actively changing lifestyle, timely screening BMD, and improving treatment compliance and so on. But several studies have found that most women have inadequate osteoporosis awareness despite being at risk of fractures.^[10,11]^ In this study we also found that most women awareness of osteoporosis was suboptimal, only about 1/3 of the subjects has a higher knowledge (31.8%). It is lower than a previous Chinese study which showed that the overall osteoporosis awareness was 67.8%.^[12]^ Education and occupation were correlated with awareness of osteoporosis, lower education level and unemployment indicated poor awareness. Although medical staff had the highest awareness score, the mean score has not reached a high level. This matches the results of previous studies.^[13,14]^ But our study did not find significant differences in the awareness of osteoporosis between different age groups and menopausal status groups. Correlation was also not been found between BMD results and awareness. For one hand it was because most women have suboptimal awareness levels, on the other hand, the subjects had a narrow age range (40–60 years).

In clinical practice, BMD is an effective tool for osteoporosis screening and fragility fracture prevention.^[15]^ Research shows that for every 1 SD reduction, fracture risk increased 1.5-fold overall.^[16]^ However, nearly one-third of women did not know that BMD was one of the methods to diagnose osteoporosis. 55.4% of women thought if they suffered from osteoporosis, they would have symptoms first, it was not too late to go to the hospital when they had any symptoms. Therefore, it is urgent to carry out health education aiming at knowledge blind spots.

While timely screening BMD is very important for prevention of osteoporosis, healthy lifestyle also plays a role and is recommended for all women at risk of osteoporosis.^[17]^ But in our study, 52.4% of women did not know that weight-bearing exercise (such as brisk walking and line dancing) could decrease bone loss. Previous study showed that bone loss was also related to decreased calcium and vitamin D intake.^[18]^ But in our study, 50.6% of the women thought it was too late to supplement calcium after menopause. In fact adequate calcium and vitamin D intake is essential to form optimal peak bone mass and lifetime maintenance.^[19]^ In addition to adequate calcium and vitamin D, estrogen therapy has been shown to successfully prevent bone loss in postmenopausal women and reduce spine and hip fracture risk by 34% in low-risk populations.^[7,20]^

There are also some shortcomings in our study. First, the subjects were recruited from our hospital only, so selection bias may exit. Therefore, a larger population with a wide range of area in the city may be considered in the future studies. Second, in this study, we only used the OPAAT questionnaire to investigate the awareness of osteoporosis in Jiaxing women, without a detailed investigation of their lifestyles. A more detailed investigation can be conducted in the future in order to help them maintain a healthy lifestyle.

## 5. Conclusions

Osteoporosis and osteopenia were highly prevalent in native Jiaxing women but their awareness of osteoporosis is inadequate. To reduce the prevalence of osteoporosis, especially among the unemployed, we should carry out effective health education through multimedia to help them to improve the awareness of osteoporosis, form a healthy lifestyle, supplement sufficient calcium tablets and vitamin D, and timely conduct BMD screening. In addition, menopause hormone therapy should also be considered for menopausal women.

## Acknowledgments

We are very thankful to all the participants for helping with the questionnaire.

## Author contributions

**Conceptualization:** Zhengfen Xu.

**Data curation:** Ying Zhou, Wei Bian.

**Formal analysis:** Huan Li.

**Investigation:** Huan Li.

**Methodology:** Wei Bian.

**Project administration:** Xiaojie Wu.

**Visualization:** Xiaojie Wu.

**Writing – original draft:** Zhengfen Xu.

**Writing – review & editing:** Zhengfen Xu, Wei Bian.
